# The association of matrix metalloproteinase-9 promoter polymorphisms with gastric cancer risk: a meta-analysis

**DOI:** 10.18632/oncotarget.20931

**Published:** 2017-09-15

**Authors:** Ziheng Peng, Jinhai Jia, Wenjian Gong, Xuehan Gao, Peiru Ma, Zhucheng Jin, Yue Fan, Yanchu Li, Xiaolin Zhang

**Affiliations:** ^1^ Department of School of Basic Medical Sciences, Hebei Medical University, Shi Jiazhuang 050017, China; ^2^ Department of Outpatient Clinic, Hebei Medical University, Shi Jiazhuang 050017, China; ^3^ Department of Epidemiology and Statistics, School of Public Health, Hebei Medical University, Shi Jiazhuang 050017, China

**Keywords:** gastric cancer, matrix metalloproteinase 9, meta-analysis, polymorphisms susceptibility risk

## Abstract

**Purpose:**

A variety of studies have observed that the single nucleotide polymorphisms (SNPs) matrix metalloproteinase-9 (MMP-9) gene may be associated with the risk of gastric cancer(GC), and a cytosine (C) to thymine (T) mutation at the -1562 site of the MMP-9 gene promoter is reported to be closely related to the susceptibility. However, because of the conflicting results of these studies, we undertook a systematic meta-analysis to assess the association between the SNPs and the risk of gastric cancer.

**Materials and Methods:**

A computerised literature search was conducted within the databases of PubMed, EMBASE, and ISI Web of Knowledge for studies on the genetic association of MMP-9-1562C/T and gastric cancer published from 2004 to 2015. The pooled odds ratio (OR) and 95% confidence intervals (CI) were estimated for each genotype using the dominant, recessive, co-dominant, and allelic models of the matrix metalloproteinase 9.

**Results:**

Our analysis indicated a significant association of MMP-9-1562C/T with gastric cancer (dominant model [CT+TT/CC]: OR = 1.121, 95% CI = 0.965–1.304; recessive model [CC+CT/TT]: OR = 1.663, 95% CI = 1.148–2.408; co-dominant model [TT/CC]: OR = 1.666, 95% CI = 1.127–2.461; [CT/CC]: OR = 1.078, 95% CI = 0.923–1.259; allelic model [T/C]: OR = 1.150, 95% CI =1.014–1.304).

**Conclusions:**

Our meta-analysis results demonstrated that MMP-9-1562C/T promoter polymorphisms increase the risk of developing gastric cancer.

## INTRODUCTION

Gastric cancer is one of the most common cancers worldwide and one of the most common causes of cancer-related deaths in the developed world (fourth for men and fifth for women). An estimated 951,600 new stomach cancer cases occurred in 2012, as well as 723,100 deaths due to stomach cancer [1]. Multiple genetic and environmental factors may contribute to the incidence of gastric cancer. When the interaction of these factors leads to a series of mutations in the related genes of the body, abnormal cells may begin hyperplasia and a tumour forms.

Matrix metalloproteinases (MMPs) are a class of proteolytic enzymes that are closely related to tumorigenesis, invasion and metastasis. Depending on their substrate specificity and domain structure, MMPs can be divided into five divergent groups: collagenases, gelatinases, stromelysins, matrilysins and membrane-type MMPs [2]. Matrix metalloproteinase-9 is a gelatinase that is located on the long arm of the human chromosome 20q13.12. Studies have found a cytosine (C) to thymine (T) mutation at the -1562 site of the MMP-9 gene promoter, which may affect the expression level of the MMP-9 gene.

Recently, several studies have revealed that promoter polymorphisms of Matrix metalloproteinase-9, may contribute to gastric cancer in humans. The results by Japanese researchers in 2005 indicated that the T allele in the MMP-9 promoter is associated with the invasive phenotype of gastric cancer [3]. Recent studies performed by Chinese researchers in 2013 [4] and by Indian researchers in 2015 [5] have confirmed this result. In addition, these Chinese researchers [4] found that the genotype of MMP-9 is correlated with TNM classification and lymph node metastasis and plays an important role in the progression and metastasis of gastric cancer. In contrast to the abovementioned investigation, two additional studies published by Chinese researchers [6, 7], one additional study published by Indian researchers [8], and one study published by Turkish researchers [9] suggest no relevant association of MMP-9 with gastric cancer. Therefore, we conducted this meta-analysis to verify the association of MMP-9 SNPs with the risk of GC.

## MATERIALS AND METHODS

### Literature and search strategy

**Figure 1:**
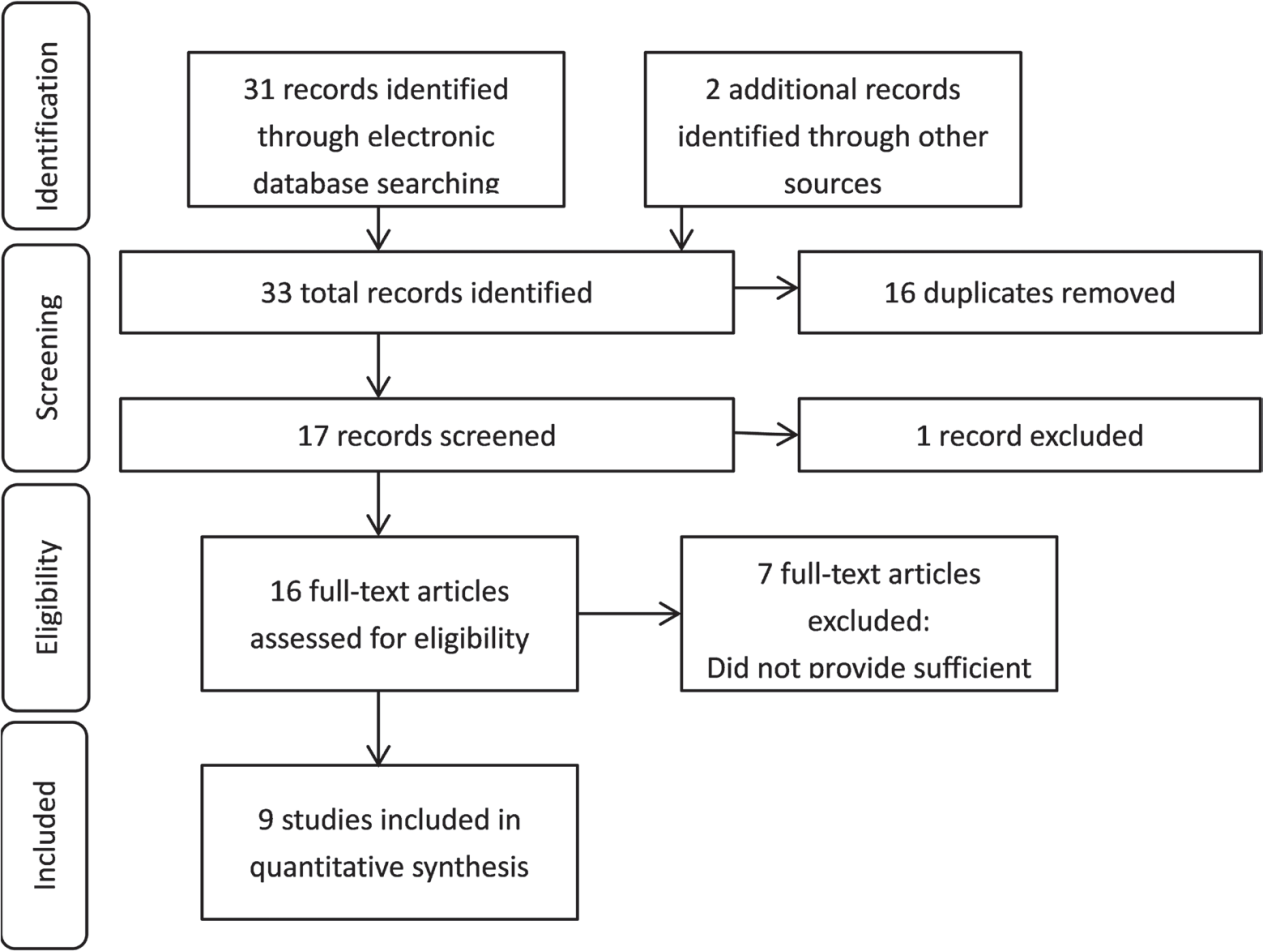
Flowchart summing the research and selection of articles for the meta-analysis

Our investigators independently conducted a systematic literature search in PubMed, Chinese National Knowledge Infrastructure (CNKI), EMBASE, WanFang (Chinese) Database, Chinese Biomedical Literature Database, and VIP (Chinese), with the last search update on January 1, 2017. We selected only studies in English and Chinese. We used the terms “(Gastric cancer) and (Matrix metalloproteinase 9)” or the equivalent Chinese terms to search the databases. In addition, we manually searched the reference lists of the included studies, as well as recent reviews, for further relevant studies (Figure 1).

### Inclusion criteria

According to the following criteria, there were nine selected eligible studies in our meta-analysis: (1) evaluation of the association between matrix metalloproteinase-9 and risk of GC; (2) report of case-control studies or nested case-control studies; (3) and reported sufficiently detailed genotype data necessary to calculate odds ratio (OR) and 95% confidence intervals (CI). Investigators applied the inclusion criteria to each study by screening its title, abstract, and full text.

### Data extraction

For each included study, the following information was collected: name of first author, year of publication, research design, geographical distribution of participants, ethnic background definitions, numbers of case and control groups, methods of DNA extraction and genotype. For studies that provided incomplete or no gene frequency data, the associated data for MMP-9 were obtained through genotype calculations. The data from each locus were divided into following two subgroups: gastric cancer and healthy control. Any dispute was resolved by discussion with all investigators.

### Statistical analysis

In this article, we performed our meta-analysis on dominant, recessive, co-dominant and alleles genotype models of the MMP-9 locus. We calculated pooled OR with a 95% CI to evaluate the association of the locus with gastric cancer. First, we conducted a *Q*-test to assess the heterogeneity of each study. Second, according to heterogeneity results, we used either the Mantel-Haenszel fixed-effects model [10] or the DerSimonian-Laird random-effects model [11] to calculate the pooled OR with a 95% CI. We considered a *P* value < 0.1 as an indicator of significant heterogeneity for the random-effects model. For the fixed-effects model, a *P* value > 0.1 was used as an indicator of significant heterogeneity. In addition, we used a funnel plot and Egger's test to assess publication bias with a *P* < 0.1 indicating statistical significance. All *P*-values were regarded as two sided. All aforementioned statistical analyses were completed using STATA version 10.0 software (STATA Corporation, College Station, TX, USA).

## RESULTS

### Characteristics of eligible studies

We chose a total of nine papers to estimate the association of MMP-9 polymorphisms with gastric cancer. The details of the research and selection process are presented in the flowchart shown in Figure 1. The gastric cancer group contained 1345 patients who had been diagnosed with gastric cardia cancer, gastric carcinoma, and gastric antrum carcinoma. The healthy population used as the control group contained 2210 patients. Therefore, 9 studies involving 1345 gastric cancer patients and 2210 healthy patients were analysed in this meta-analysis (Table 1).

**Table 1:** Main characteristics of all eligible studies selected in the meta-analysis

First author	Publication year	Ethnicity	Number		NOS
			Cases^a^	Control^a^	
Chen Jian [3]	2013	Chinese	98	100	7
Zhang Weiqiang [6]	2007	Chinese	170	200	6
Zhang Xuemei [5]	2004	Chinese	228	774	7
Shunji Matsumura [2]	2005	Japanese	177	224	8
SugreevVerma [4]	2015	Indian	230	233	7
NiluferAvci [8]	2015	Turkey	79	65	7
D.Krishnaveni [7]	2012	Indian	132	132	7
Ji Hye Kim [13]	2011	Asian	152	313	8
FjgmKubben [12]	2006	European	79	169	7

^a^Gastric cancer/health

In addition, we evaluated the quality of the included studies according to the Newcastle-Ottawa Scale (NOS) [12]. This scale was used to access the studies on three criteria: determination of either the exposure or the outcome of interest (0–3 points), comparability of the groups (0–2 points) and selection of the study groups (0–4 points). The best possible score of this scale is 9 points. In this meta-analysis, two studies [3, 13] scored 8 points; six studies [4–6, 8, 9, 14] scored 7 points; and one study [7] scored 6 points.

### Association between MMP-9 polymorphism and GC susceptibility

**Figure 2:**
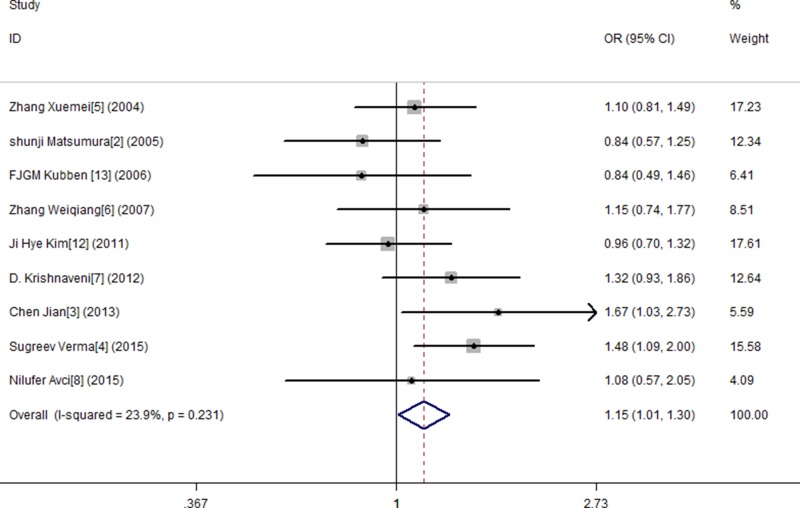
Meta-analysis forMMP9-1562 C/T polymorphism and gastric cancer susceptibility in allele genetic model

**Figure 3:**
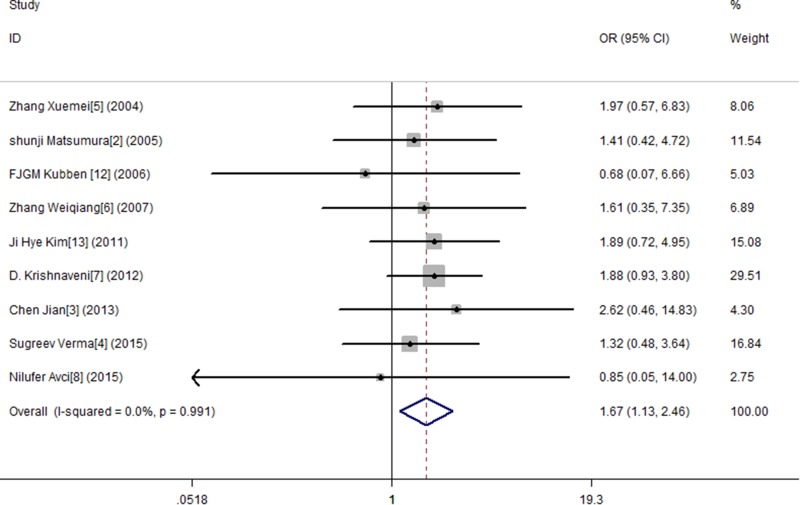
Meta-analysis for MMP9-1562 C/T polymorphism and gastric cancer susceptibility in co-dominant genetic model (TT)

**Figure 4:**
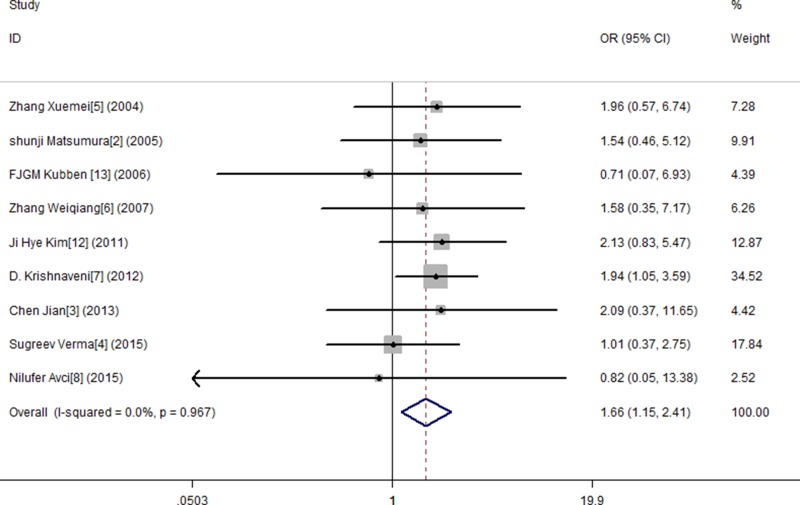
Meta-analysis for MMP9-1562 C/T polymorphism and gastric cancer susceptibility in recessive genetic model

**Figure 5:**
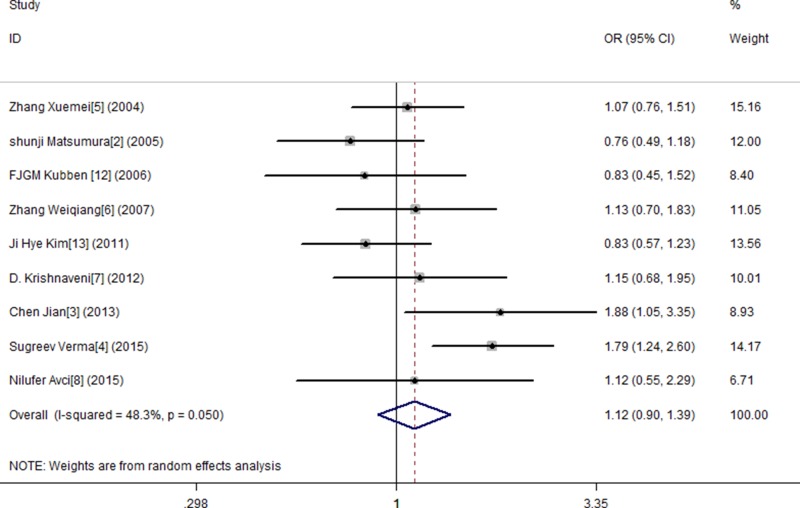
Meta-analysis for MMP9-1562 C/T polymorphism and gastric cancer susceptibility in dominant genetic model

**Figure 6:**
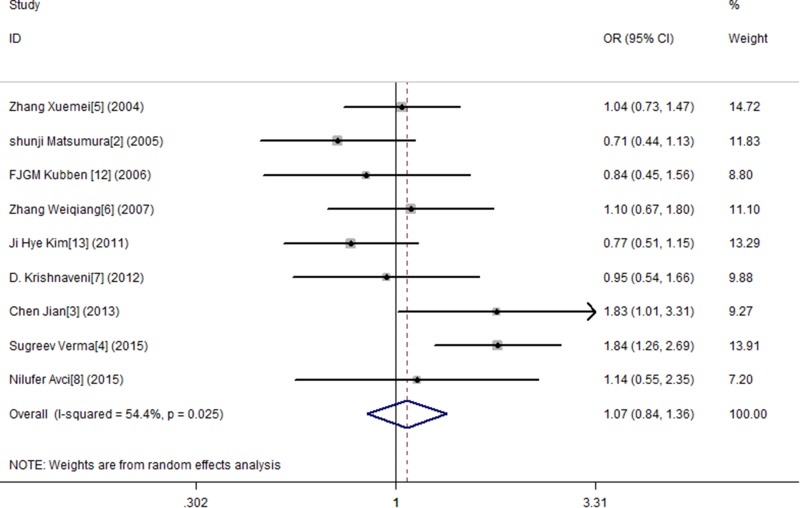
Meta-analysis for MMP9-1562 C/T polymorphism and gastric cancer susceptibility in co-dominant genetic model (CT)

In this study, by conducting the combined analysis, we observed a statistical divergence between SNP MMP-9 and risk of GC in all genetic models (Table 2). The T allele of MMP-9 was significantly associated with GC, demonstrating a pooled OR of 1.150 (95% CI = 1.014–1.304) (Figure 2). The TT genotype showed approximately a 1.666-fold (OR = 1.666, 95% CI = 1.127–2.461) increased risk of GC compared with the MMP-9 CC genotype (Figure 3). The results from the recessive genotype models showed similar conclusions (OR: 1.663, 95% CI = 1.148–2.408) (Figure 4). However, the MMP-9 dominant genotype models showed no relationship with GC (OR = 1.121, 95% CI = 0.965–1.304) (Figure 5). The results from CT genotype models showed similar conclusions (OR = 1.078, 95% CI = 0.923–1.259) (Figure 6).

**Table 2:** Main results of pooled ORs in the meta-analysis (comparison between patients with gastric cancer and healthy population)

Genetic model		OR (95% CI)	*P* heterogeneity
Co-dominant model	CC	1	
	CT	1.072 (0.843, 1.362)^b^	0.025
	TT	1.666 (1.127, 2.461)^a^	0.991
Dominant model	CT+TT vs. CC	1.119 (0.899, 1.392)^b^	0.050
Recessive model	CC+CT vs. TT	1.663 (1.148, 2.408)^a^	0.967
Allele	C vs. T	1.150 (1.014, 1.304)^a^	0.231

^a^Fixed-effects model was constructed.

^b^Random-effects model was constructed.

### Publication bias

**Figure 7:**
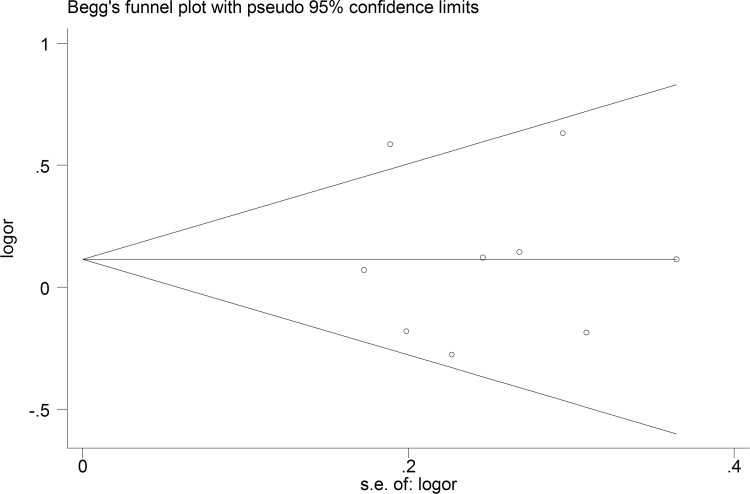
Funnel plots of the MMP-9 polymorphism studies: dominant model No obvious funnel asymmetry was observed. Egger's test of the value from the funnel plot showed no statistical significance (*p* = 0.917).

We employed the funnel plot and Egger's test to evaluate any potential publication bias. The shape of the funnel plots suggested no evident publication bias for MMP-9 among the co-dominant, dominant and recessive genotype models. A *P* value > 0.1 for Egger's test further verified these results (Figure 7).

## DISCUSSION

The matrix metalloproteinases (MMPs) family comprises more than 20 enzymes and plays critical roles in cellular apoptosis, angiogenesis, tumour growth and metastasis. As essential regulators of the microenvironment of the cell, MMPs are capable of degrading extracellular matrix (ECM) by cleaving it, which is considered as a barrier in cellular invasion [15]. The ECM of the gastric mucosa is composed of a number of macromolecules, such as collagen, laminin, proteoglycan, elastin, fibronectin and hyaluronic acid, and their degradation by MMPs play an important role in maintaining the cellular microenvironment [16]. The activity of MMPs is modulated by transcriptional regulation and their interaction with tissue inhibitors of metalloproteinases (TIMPs). MMPs and TIMPs play a key role in several steps of tumour dissemination and metastasis [17]. Expression of MMP genes can vary and affect the balance between synthesis and degradation of ECM proteins, which may contribute to the inter-individual diversity of susceptibility to many complex diseases, including cancer [18].

Alveolar macrophages, polymorph nuclear leukocytes, osteoclasts and malignant cells primarily express MMP-9 [19], which is also known as gelatinase B due to its ability to degrade collagen type IV, collagen type V and elastin. These cells secrete MMP-9 in an inactivated form as a 10 kDa propeptide, and other MMPs or tissue plasminogen activator (tPA) plasmin system activate them [20, 21]. Additionally, under pathological conditions including gastrointestinal inflammation and gastric cancer, MMP-9 can be derived from stromal cells, such as inflammatory cells and fibroblasts [22, 23], and enhanced level of MMP-9 has been described. Furthermore, in vivo studies in MMP-9 deficient mice found that implanting MMP-9 expression in the bone marrow enhanced tumour metastasis, which could facilitate cancer cell migration by promoting angiogenesis [5].

MMP-9-1562C/T is one of the polymorphism positions in the MMP-9 gene promoter contig sequence. A cytosine (C) to thymine (T) transition at nucleotide -1562 in the promoter region of the MMP-9 gene generates low activity for C/C and high activity for C/T and T/T genotypes in gene transcription [5]. The latter activities are confirmed by transient transfection experiments and DNA–protein interaction assays, which indicate that due to preferential binding of a putative transcription repressor protein to the C allelic promoter the T allele had a higher promoter activity than the C allele [24].

The activity of increasing MMP-9 could be downregulated by many extracellular factors, such as MMP-1, -3, -7, -10, -26, trypsin-2 and neutrophil elastase [25]. Among these, the most important endogenous inhibitor of MMP-9 is the tissue inhibitors of metalloproteinases 1 (TIMP-1), which blocks the cleavage effect of MMP-9 extracellularly. However, several studies have revealed that TIMP-1 also inhibits the membrane-protein shedding process and the cell signal regulatory effect of MMP-9 [26–28]. Because of its special properties, initially TIMP-1 was considered a tumour suppressing gene. Later, researchers found that TIMP-1 also functions independently of MMP-9 to promote tumour growth and inhibit apoptosis [29].

Some studies have found a protective effect against digestive cancers [30] and lung cancer [31] for MMP-9 polymorphism, but its effects in prostate cancer vary [32]. Recently, some results [33] have provided new evidence that the imbalance of MMP-9/TIMP-1 is one of the regulation mechanisms to promote tumourigenicity and metastasis of prostate cancer cells. However, other researchers hold a different opinion and the causal mechanism of this imbalance remains unclear. In addition, in the past two years, an increasing number of studies have revealed the relationship between MMP-9 and oesophageal squamous cell carcinoma [34], ovarian cancer [35], nasopharyngeal carcinoma [36, 37], Ewing sarcoma [38], and bladder cancer [39]. Different microenvironments and tissue interactive mechanisms may lead to the presence of the divergence. By conducting studies with larger sample sizes and better consideration of environmental factors, researchers can make progress in determining the cariogenic effect of these polymorphisms.

The present study had some limitations that must be discussed. First, although we collected all eligible studies, the number of studies was small, which meant that analyses may not have had enough statistical power to explore the association of these polymorphisms with cancer susceptibility. As we all known, any interpretation of meta-analysis must be bounded within its limited context, and thus, only further and larger-scaled studies can lessen the likelihood of type I and type II errors. Second, the lack of available information about adjusted ORs by age, gender, smoking status and different histological types of gastric cancer, especially diets and alcohol consumption, prevented a more precise evaluation. Objectively speaking, it is necessary to evaluate the roles of those special environmental and lifestyle factors. Third, we cannot ignore the possible existence of gene–gene interactions. Ideally, we should evaluate the gene only after determining the roles of MMPs’ inhibitors, such as TIMP-1, TIMP-2, TIMP-3, and TIMP-4, in the development of gastric cancer. Moreover, some polymorphisms may play different roles in different tissues among people with different genetic backgrounds. A lack of data on ethnicity analysis prevented us from conducting a subgroup analysis on different populations. In spite of this, our meta-analysis has several strengths. First, our results arise from an analysis that combined the data of several studies rather than evaluating the studies independently. This significantly enhanced the statistical power of the analysis and helped us to reach a more reliable conclusion. Second, we tested publication bias and detected none, which indicates a minimised chance of misleading results.

In conclusion, the results of our meta-analysis demonstrated that promoter region polymorphisms of the MMP-9 gene were associated with the susceptibility of gastric cancer. More comprehensive studies may eventually result in a better understanding of the association of MMP-9-1562C/T polymorphisms with gastric cancer.
